# Impact of microvessel patterns and immune status in NSCLC: a non-angiogenic vasculature is an independent negative prognostic factor in lung adenocarcinoma

**DOI:** 10.3389/fonc.2023.1157461

**Published:** 2023-04-26

**Authors:** Erna-Elise Paulsen, Sigve Andersen, Mehrdad Rakaee, Mona Irene Pedersen, Ana Paola Lombardi, Mette Pøhl, Thomas Kilvaer, Lill-Tove Busund, Francesco Pezzella, Tom Donnem

**Affiliations:** ^1^ Department of Pulmonology, University Hospital of North Norway, Tromso, Norway; ^2^ Department of Oncology, University Hospital of North Norway, Tromso, Norway; ^3^ Institute of Clinical Medicine, UiT The Arctic University of Norway, Tromso, Norway; ^4^ Department of Molecular Pathology, University Hospital of North Norway, Tromso, Norway; ^5^ Institute of Medical Biology, UiT The Arctic University of Norway, Tromso, Norway; ^6^ Department of Oncology, Copenhagen University Hospital, Rigshospitalet, Copenhagen, Denmark; ^7^ Department of Clinical Pathology, University Hospital of North Norway, Tromso, Norway; ^8^ Nuffield Department of Clinical Laboratory Sciences, University of Oxford, John Radcliffe Hospital, Oxford, United Kingdom

**Keywords:** non-angiogenic tumors, angiogenesis, immunology, immune cells, vessel co-option

## Abstract

**Introduction:**

Non-small cell lung carcinomas (NSCLC) exhibit different microvessel patterns (MVPs). Basal (BA), diffuse (DA) and papillary (PA) patterns show signs of angiogenesis (new blood vessels), while an alveolar pattern indicates that tumors are co-opting existing normal vessels (non-angiogenic alveolar, NAA). NAA tumor growth is known to exist in NSCLC, but little is known about its prognostic impact in different histological subgroups, and about associations between MVPs and immune cell infiltration.

**Methods:**

Detailed patterns of angiogenic and non-angiogenic tumor growth were evaluated by CD34 immunohistochemistry in whole tissue slides from 553 surgically treated patients with NSCLC stage I-IIIB disease. Associations with clinicopathological variables and markers related to tumor immunology-, angiogenesis- and hypoxia/metabolism were explored, and disease-specific survival (DSS) was analyzed according to histological subtypes.

**Results:**

The predominant MVP was angiogenic in 82% of tumors: BA 40%, DA 34%, PA 8%, while a NAA pattern dominated in 18%. A contribution of the NAA pattern >5% (NAA+), i.e., either dominant or minority, was observed in 40.1% of tumors and was associated with poor disease-specific survival (DSS) (*p*=0.015). When stratified by histology, a significantly decreased DSS for NAA+ was found for adenocarcinomas (LUAD) only (*p*< 0.003). In multivariate analyses, LUAD NAA+ pattern was a significant independent prognostic factor; HR 2.37 (CI 95%, 1.50-3.73, *p*< 0.001). The immune cell density (CD3, CD4, CD8, CD45RO, CD204, PD1) added prognostic value in squamous cell carcinoma (LUSC) and LUAD with 0-5% NAA (NAA-), but not in LUAD NAA+. In correlation analyses, there were several significant associations between markers related to tumor metabolism (MCT1, MCT4, GLUT1) and different MVPs.

**Conclusion:**

The NAA+ pattern is an independent poor prognostic factor in LUAD. In NAA+ tumors, several immunological markers add prognostic impact in LUSC but not in LUAD.

## Introduction

Angiogenesis is the process by which new blood vessels are formed. The induction of angiogenesis, in order to ensure sufficient supply of oxygen and nutrients in growing tumors, was one of the original Hallmarks of cancer (1). However, in the recent update “Hallmark of cancer: new dimensions” by Hanahan, “angiogenesis” was finally modified to “inducing or accessing vasculature” (1–3). After years of increasing reports and a long-lasting debate in the cancer community, there is now an appreciation that tumors can become sufficiently vascularized either by “switching on” angiogenesis or by co-opting normal tissue vessels (2). We and others have previously reviewed convincing data concluding that many tumors, partly or completely, grow without angiogenesis (4–6). Non-angiogenic tumors may also be a cause of resistance to anti-angiogenic therapy (4–6).

Non-small cell lung cancer is a common and deadly disease (7). An emerging understanding of the tumor immune microenvironment, and the introduction of immunotherapy, has led to improved outcomes for many non-small cell lung cancer (NSCLC) patients (8). Yet, the majority of patients progress due to resistance mechanisms. There is a complex interplay between tumor blood supply and tumor immunity, and potential clinical implications are being explored, including treatments combining angiogenesis inhibitors and immunotherapy (9, 10). Hence, there is an increasing need for knowledge of the biology of this interplay, and of the significance of different subtypes of angiogenic or non-angiogenic vessel patterns.

Along with kidneys, liver and brain, lungs are extensively vascularized and non-angiogenic tumor growth is frequently reported (4, 5). In NSCLC, early studies have demonstrated that as many as 10-30% of tumors grow in a non-angiogenic fashion (4, 5, 11). NSCLC data also indicate that an angiogenic microvessel pattern (MVP) may induce a more extensive immunological response when compared to a non-angiogenic MVP, while tumors with co-opted blood supply were associated efficient mitochondrial metabolism (4, 5, 12).

Lung adenocarcinomas (LUAD) and squamous cell carcinomas (LUSC) may be considered two different entities, distinctive both in biological characteristics and treatment approaches. We wanted to explore the prognostic impact of MVPs stratified by histology, and whether combinations with immunological markers added prognostic impact. We have previously explored multiple markers related to angiogenesis, hypoxia/metabolism and tumor immunology in the same cohort. Herein we wanted to examine associations between MVPs and these markers, with emphasis on immunological markers representing essential innate and adaptive immune cells (13–26).

## Material and methods

### Study design and patients

Tumor specimens from consecutive patients undergoing radical resection for NSCLC, pathological stage I to IIIB, at the University Hospital of North Norway (UNN, n=295) and the Nordland Hospital (NH, n=258) from 1990 through 2010, were collected retrospectively. In total, 553 patients with complete medical records were included, as previously reported (20). Follow-up was completed on October 1, 2013, and median follow-up of survivors was 86 months (range, 34-267 months).

Tumors were restaged according to the 8th International Union Against Cancer TNM classification, and histologically re-evaluated according to the 2015 WHO classification (27, 28). Patients with missing or inadequate tissue quality on stained slides (n=69) were excluded, leaving 484 patients available for analyses. Each tumor was represented by a single whole slide image, and adjacent hematoxylin and eosin-stained sections were available for morphologic review.

The study was approved by the Norwegian Data Protection Authority and the Regional Committee for Medical and Health Research Ethics (protocol ID, 2011/2503). Clinicopathologic variables, survival data and biomarker expressions are reported in accordance with the REMARK (reporting recommendations for tumor marker prognostic studies) guidelines (29).

### Immunohistochemistry CD34

IHC staining was performed using the Discovery Ultra autostainer (Ventana, Roche, Tucson, AZ) on formalin-fixed paraffin-embedded whole tissue sections. The slides were deparaffinized in the instrument (68°C, 3 cycles á 12 min). Antigen retrieval was applied using ULTRA Cell Conditioning-1 (Ventana, Roche) for 32 minutes at 95°C. The sections were incubated for 32 minutes with IVD primary mouse monoclonal antibody anti-CD34 (clone QBEnd/10; cat# 790-2927; Ventana, Roche). As a secondary antibody, OmniMap anti-Mouse HRP (cat# 760-4310, Ventana, Roche) was loaded for 16 minutes, followed by 4 minutes of HQ HRP amplification (cat# 760-052, Ventana, Roche). The detection chromogen used was the DAB kit (cat#760-159; Ventana, Roche) with 32 minutes of incubation. Counterstaining was performed using hematoxylin II (cat#790-2208, Ventana, Roche) for 12 minutes and then loading bluing reagent for 4 minutes.

Slides were digitized using a Pannoramic 250 Flash III (3DHistech, Budapest, Hungary), slide scanner.

### Markers of tumor immunity, angiogenesis and hypoxia/metabolism

We have previously reported the distribution and prognostic impact of >100 markers of angiogenesis, immunology, hypoxia/metabolism and EMT expressed in the NSCLC tumor microenvironment, in a tissue microarray (TMA) material in the same patient cohort. Markers were analyzed by immunohistochemistry or *in situ* hybridization and scored in a manual, semi-quantitative or digital, quantitative fashion, as previously published (13–18, 20–24, 30–49). In the present study, we included data for the most relevant markers involved in tumor immunity (n=22), angiogenesis (n=30) and hypoxia/metabolism (n=14) (**Supplementary Table S1**). Markers were evaluated in both stromal (_S) and tumor epithelial (_T) compartments, separately or combined (_TS). Initially markers were analyzed in a TMA material representing a cohort of 335 patients surgically treated between 1990 and 2005. Additional analyzes, including most immunology markers, followed a cohort expansion by 218 patients treated between 2005 and 2011 (553 in total).

### Microvessel pattern evaluation

**Figure 1:**
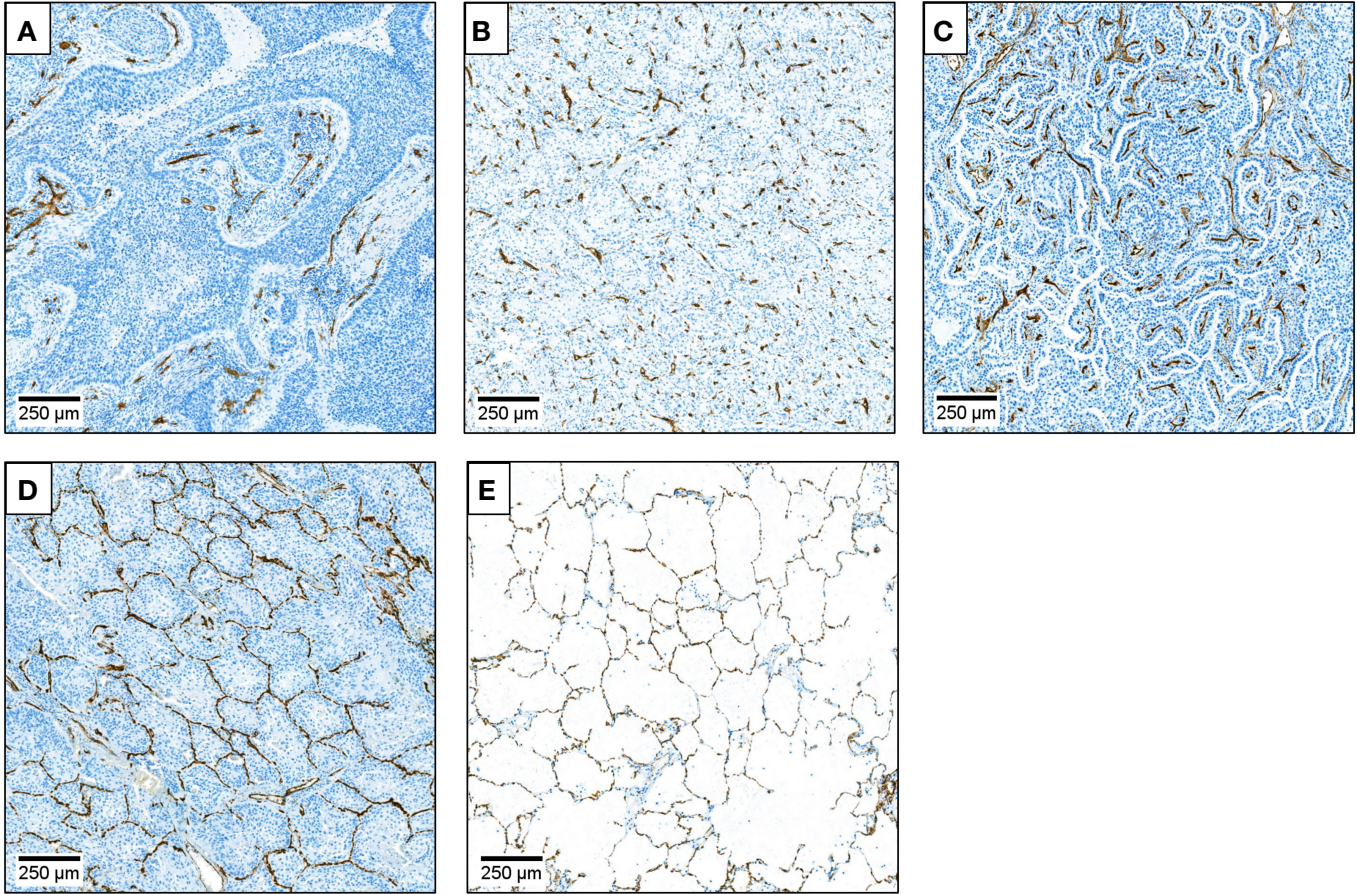
Description of microvessel patterns (MVPs) and their quantification. Microvessels were identified by CD34 IHC DAB staining (brown) of endothelial cells. **(A)** Basal angiogenic (BA) microvessel pattern (MVP). Tumor cells are arranged in epithelial nests surrounded by connective tissue. Most vessels are in the connective tissue beneath the neoplastic epithelium. **(B)** Diffuse angiogenic (DA) MVP. Normal lung architecture is diffusely replaced. New vessels, stroma and neoplastic cells are mixed and without recognizable architectural structure. **(C)** Papillary angiogenic (PA) MVP. Vessels are present in a stromal stalk covered by neoplastic cells, mostly as a monolayer. Normal lung architecture is recognizable in some areas. Remodeling of the alveolar structure occurs. **(D)** Non-angiogenic alveolar (NAA) MVP Vessels arise from alveolar septa only, normal lung tissue architecture is preserved (“chicken wire”). Tumor cells grow in a solid fashion filling alveolar spaces. No endothelial or stromal cells present among neoplastic cells. Anthracitic pigment may be trapped in the parenchyma. **(E)** Normal lung. The contribution of each of the four different MVPs was quantified as a percentage of the tumor tissue represented in the whole slide image, estimated in 5% increments.

All tissue slides were digitally reviewed by an experienced pathologist (FP) and an oncologist (EP) using QuPath software v.0.1.2 (Queen’s University, Belfast, Northern Ireland) (50). Microvessel patterns (MVPs) were identified by CD34 IHC staining and quantified as a percentage of the tumor tissue represented in the whole slide image. The MVPs were defined as angiogenic, including the basal (BA), diffuse (DA) and papillary (PA) subtypes, or non-angiogenic alveolar (NAA), in accordance with Pezzella et al, 1997 (11). Detailed descriptions of the MVPs are described in **Figure 1**. The contribution of each of the four MVPs was manually estimated in 5% increments; all MVPs could be represented in each tumor.

### Statistical methods

Statistical analyses were performed using IBM SPSS Statistics (Version 29) or RStudio with R version 4.1.2 (utilizing packages survival, surminer, ggplot, dplyr, tidyverse, ggcorrplot, ggstatplot). The χ^2^ test or Fischer’s exact test was used to investigate the associations between clinicopathological variables and MVPs. Associations between MVPs and other markers (n=123) were evaluated by Spearman’s rank correlation (all non-parametric variables). The contribution of each MVP (continuous, interval variable) was tested against the mean value (categorical, ordinal ranked variable) of all markers. To adjust for multiple testing, Bonferroni correction was conducted. Disease-specific survival (DSS) was defined as the time from surgery to lung cancer death. Univariate analysis of survival according to MVPs was evaluated by the Kaplan-Meier method. The log-rank test was applied to assess statistically significant differences between survival curves. For survival analyses, the cutoff for dichotomization of MVP variables was selected by applying an “optimal *p-*value” approach. Cox proportional hazards ratios were calculated. Multivariate analysis included relevant covariates including, but not limited to, variables found significant in univariate analyses. The backward conditional method was used for model fitting. Probability for stepwise entry and removal was set at 0.05 and 0.10, respectively. For statistical tests, a significance level <0.05 was considered statistically significant.

## Results

### Patient characteristics

Demographic, clinical, and histopathologic variables and their associations with DSS are presented in **Table 1**. The median age was 67 years (range, 28-85), 33% were female. Seventy-six patients (14%) received postoperative radiotherapy because of pN2 disease or nonradical surgical margins. Following its introduction into Norwegian national guidelines in 2005, 43 patients (8%) received adjuvant systemic therapy. None of the patients received immune checkpoint inhibitors or antiangiogenic treatment during follow-up. Clinicopathological variables did not differ significantly between patients included in the MVP analysis (scored) and patients where no adequate tissue was available (missing), except for frequencies of pneumonectomy and vascular infiltration (24 vs 36% and 16 vs 29%).

**Table 1:** Clinicopathological variables and non-angiogenic (NAA) microvessel pattern (MVP) as predictors of disease-specific survival in resected NSCLC patients and in LUSC and LUAD histological subgroups (univariate analyses; log rank test; unadjusted proportional hazard ratios).

	All patients					LUSC					LUAD				
	N (%)	5 year (%)	Median (m)	HR (95% CI)	*p*	N	5 year (%)	Median (m)	HR (95% CI)	*P*	N	5 year (%)	Median (m)	HR (95% CI)	*P*
**Age**					.64					.93					.65
<65	231 (42)	58	127	1		111	64	235	1		115	52	71	1	
≥65	322 (58)	59	NR	.94 (.72-1.22)		196	63	NR	1.02 (.69-1.50)		124	52	NR	.92 (.63-1.33)	
**Sex**					**.025**					.097					**.028**
female	180 (33)	64	189	1		77	72	NR	1		100	59	189	1	
male	373 (67)	55	91	1.39 (1.04-1.86)		230	61	235	1.49 (.93-2.37)		139	46	57	1.54 (1.05-2.26)	
**Smoking**					.07					.20					.18
former	182 (33)	52	84	1		103	60	114	1		76	41	47	1	
present	350 (63)	62	235	1.14 (.61-2.15)		196	67	235	1.50 (.54-4.19)	.44	150	57	NR	.93 (.42-2.08)	.86
never	21 (4)	50	21	.75 (.57-.98)		8	42	19	.75 (.51-1.12)	.16	76	54	190	.70 (.47-1.03)	.072
**ECOG perf. status**					**.011**					.10					.052
0	324 (59)	63	235	1		168	68	235	1		153	57	NR	1	
1	191 (34)	52	70	1.51 (1.15-1.99)		115	59	114	1.52 (1.03-2.25)		74	43	51	1.54 (1.05-2.27)	
2	38 (7)	52	NR	1.46 (.82-2.59)		24	59	NR	1.43 (.65-3.14)		12	38	25	1.87 (.81-4.33)	
**Weightloss**					.96					.92					.85
N	497 (89)	58	189	1		272	63	235	1		221	52	73	1	
Y	55 (10)	59	NR	1.01 (.64-1.60)		34	64	NR	.97 (.50-1.86)		18	52	98	1.07 (.54-2.11)	
Missing	1 (1)														
**Surgical procedure**					**<.0001**					**.005**					**<.0001**
wedge	22 (4)	78	NR	1		8	53	NR	1		13	92	NR	1	
lobectomy	389 (70)	63	189	2.23 (.83-6.02)		201	71	235	.91 (.29-2.89)		182	55	104	6.78 (.94-48.7)	
pneumonectomy	142 (26)	42	30	4.38 (1.60-11.99)		98	49	35	1.72 (.53-5.54)		44	25	25	18.01 (2.46-132.91)	
**pN status**					**<.0001**					**<.0001**					**<.0001**
N0	379 (69)	70	235	1		213	74	235	1		160	64	189	1	
N1	118 (21)	36	35	2.86 (2.12-3.87)		77	42	29	3.28 (2.19-4.93)		41	29	37	2.41 (1.52-3.81)	
N2	56 (10)	23	21	4.28 (2.96-6.18)		17	21	14	6.03 (3.28-11.09)		38	22	24	3.45 (2.14-5.56)	
**pT status**					**<0.0001**					**<.001**					***<.001***
T1	180 (32)	72	235	1		89	74	235	1		89	70	189	1	
T2	208 (38)	54	83	1.87 (1.33-2.63)		112	65	NR	1.64 (.99-2.71)		92	44	47	2.15 (1.35-3.42)	
T3	104 (19)	56	NR	1.69 (1.12-2.54)		70	63	NR	1.53 (.87-2.72)		34	43	50	2.08 (1.15-3.76)	
T4	61 (11)	31	21	3.46 (2.26-5.30)		36	35	17	3.59 (1.98-6.52)		24	23	25	3.70 (2.00-6.85)	
**pStage**					**<.0001**					**<.0001**					**<.0001**
Stage I	232 (42)	74	235	1		118	75	235	1		110	72	189	1	
Stage II	185 (34)	59	114	1.70 (1.22-2.38)		121	71	NR	1.40 (.86-2.25)		63	43	47	2.23 (1.39-3.56)	
Stage IIIA	136 (25)	28	21	4.14 (2.99-5.75)		68	30	16	4.51 (2.81-7.25)		66	22	25	3.93 (2.48 -6.22)	
**Histological subgroups**					.24										
LUSC	307 (56)	64		1											
LUAD	239 (43)	52		1.25 (.96-1.63)											
Other	7 (1)			.95 (.24-3.86)											
**Differentiation**					**<.001**					**.008**					**.008**
Well	82 (15)	72	NR	1		34	72	NR	1		48	72	NR	1	
Moderate	240 (43)	63	189	1.61 (1.00-2.59)		154	71	235	1.27 (.60-2.68)		84	50	57	2.18 (1.17-4.08)	
Poor	231 (42)	49	51	2.41 (1.51-3.83)		34	52	71	2.19 (1.04-4.62)		107	44	50	2.61 (1.43-4.76)	
**Vascular infiltration**					**<.0001**					**.011**					**.003**
No	453 (82)	62	235	1		245	67	235	1		202	56	104	1	
Yes	97 (17)	38	89	1.94 (1.42-2.66)		62	49	39	1.77 (1.41-2.74)		34	25	30	2.18 (1.38-3.56)	
Missing	3 (1)														
**Radiacal resection**					.11					.28					.067
R0	506 (92)	59	190	1		274	65	235	1		225	53	83	1	
R1/R2	47 (8)	47	57	1.43 (.93-2.20)		33	56	114	1.35 (.79-2.34)		14	17	47	1.96 (.95-4.03)	
**NAA microvessel pattern**					**.015**					.66					**.003**
NAA- (0-5%)	290 (52)	235	66	1		181	66	235	1		104	64	NR	1	
NAA+ (>5%)	194 (35)	77	52	1.42 (1.07-1.90)		81	65	NR	.90 (.57-1.43)		111	45	50	1.88 (1.24-2.85)	
Missing	69 (13)														

bold numbers are significant.

CI, confidence interval; HR, hazard ratio; DSS, disease-specific survival; ECOG perf. status, Eastern Cooperative Oncology Group performance status; LUAD, lung adenocarcionma; LUSC, lung asquamous cell carcinoma; N, number; NAA, non-angiogenic alveolar; NR, not reached; NSCLC, non-small cell lung carcinoma; pN status, pathological nodal stage; pT status, pathological tumor stage; y, year.

### Microvessel pattern distribution

We observed two or more MVPs in 316 of the 484 tumors. In the 170 cases where a single pattern covered the entire tumor, an angiogenic pattern was more frequently found (BA n=79, DA n=65, PA n=11) than a non-angiogenic (NAA n=15). Any presence (5-100% of the tumor area) of the BA, DA, PA, and NAA MVPs was observed in 257, 274, 68 and 260 of the 484 tumors, respectively. The predominant MVP in each tumor represented from 40% (in tumors with multiple MVPs) to 100% of the area. The predominant MVP was of an angiogenic subtype in 82% (BA 40%, DA 34%, PA 8%) of cases, while the NAA pattern was predominant in 18% of tumors. The distribution of MVPs in tumors is presented in detail **Supplementary Figure S1**.

### Correlations analyses

Correlation matrices presented in **Supplementary Figure S2** summarize all correlations between MVPs and markers related to immunology, angiogenesis and hypoxia/metabolism. After Bonferroni correction for multiple testing, *p*-values <.0004 were considered significant. Correlation coefficients >.2 were regarded as relevant in this explorative setting. A selection of significant correlations (*p*<0.01) between MVPs and included markers are presented in **Table 2**.

**Table 2:** Correlations between MVP and other markers.

Correlation coefficient	Basal angiogenic (BA)	Diffuse angiogenic (DA)	Papillary angiogenic(PA)	Non-angiogenic alveolar (NAA)
Positive >0.3	**GLUT1_T (.426, p=2.1x10^-13^)** **MCT1_T (.340, p=3.4x10^-8^)**			**MVD_T (.308, p=1.8x10^-7^)**
Positive 0.2 - 0.3	**CD138_T (.298, p=4.8x10^-7^)** **CD138_S (.293, p=7.5x10^-7^)** miR210_T (.222, p=.0006)	**CSF1R_T (.231, p=.0001)**	**FGFR1_T (.210 p=.00040)** LDH5_T (.201, p=.0009)	**MVD_S (.215, p=.0003)**
Positive 0.1 - 0.2	PDGFB_T (.161, p=.007) CAIX_T (.172, p=.005) MCT4_S (.193, p=.002)	CD3_T (.128, p=.007)* CD3_TS (.147, p=.002)* CD4_TS (.130, p=.005)* CD8_TS (.165, p=.00037)* PD1_T (.131, p=.005)* PDL1_T (.171, p=.0002)* CD163_T (.165, p=.001)* CD204_T (.161, p=.001)* CD68_T (.141, p=.004)* CSF1R_S (.169, p=.005)	CD1A_S (.165, p=.006) MVD_T (.163, p=.007) Notch4_T (.158, p=.009)	
Negative -0.1 - -0.2	CD3_T (-.139, p=.003)* PDL1_T (-.181, p=.0001)* CD163_T (-.143, p=.005)* CD204_T (-.155, p=.002)* CSF1R_T (-.196, p=.001) CD1A_T (-.162, p=.007) MVD_S (-.163, p=.007) DLL4_T (-.164, p=.007) miR21_T (-.171, p=.005)	CD138_T (-.189, p=.002) CD138_S (-.157, p=.009)	PDL1_S (-.125, p=.007)* CD163_S (-.144, p=.002)* CSF-1R_S (-.179, p=.003)	CD8_S (-.134, p=.004)* VEGFA_S (-.167, p=.005) VEGFR3_S (-.184, p=.002) Tie2_S (-.171, p=.005)
Negative -0.2 - -0.3	MVD_T (-.210, p=.00044) **MCT4_T (-.268, p=.00001)**	**GLUT1_T (-.219, p=.0003)**	**GLUT1_T (-.282, p=.000002)**	CSF1R_S (-.208, p=.0005) CD138_S (-.211, p=.00042) PDGFRβ_S (-.205, p=.00001)* **MCT1_T (-.232, p=.0002)** **MCT4_S (-.247, p=.00007)**
Negative <-0.3	**FGFR1_T (-.307, p=1.6x10^-7^)**		**MCT1_T (-.339, p=4.0x10^-8^)**	

*n=553 (expanded cohort); others: n=335 (original cohort). T, tumor; _S, stroma; _TS, tumor and stroma.

All correlations included were found significant to a p<0.01 level. Bold: Significant after Bonferroni correction for multiple testing. Spearman’s ranked correlations between MVPs and other markers.

#### Correlations with markers related to tumor immunity

Few immune markers were significantly correlated with MVPs. CD138 (plasma cells) in tumor and stroma was positively correlated with BA (.293-.298). For DA MVP, positive correlations with CSF1R were found (monocyte and myeloid dendritic cells) (.231).

#### Correlations with markers related to angiogenesis

A previous assessment of microvessel density, also by CD34 staining, showed a positive correlation with NAA (_T:.308, _S:.215). Except for PDGFRβ_S (-.205 in NAA) and FGFR1_T (-.307 in BA and.210 in PA), markers of angiogenesis (VEGF-A, -C, -D, VEGFR-1, -R2, -R3, PDGF-A, -B, -C, -D, PDGFR-α, bFGF, Notch1, -4, Jagged1, DLL4, Ang1, -2, -4, Tie2), were not significantly correlated with MVPs.

#### Correlations with markers related to hypoxia and metabolism

Markers involved in hypoxia and metabolism were divergently expressed in different MVPs. A significant positive correlation with the BA MVP was found for MCT1_T (.340), and GLUT1_T (.426), and a negative correlation for MCT4_T (-.268). Oppositely, significant negative correlations were found for GLUT1_T in PA (-.282), MCT4_S in NAA (-.247) and for MCT1_ in both PA (-.339) and NAA (-.232).

### Associations with clinicopathological variables

Tumors with a predominant BA MVP were significantly associated with male sex and LUSC, and the NAA pattern with female sex and with LUAD histology. No other associations with other clinicopathological variables were found.

### Univariate survival analyses

Pathological TNM stage, T- and N-stage, tumor differentiation, vascular infiltration, surgical procedure, sex and ECOG status were significant prognostic indicators for DSS in all patients (all *p*< 0.001) (**Table 1**).

The angiogenic MVPs (BA, DA or PA) were not significantly associated with DSS, when analyzed separately as dichotomized variables (none vs any for BA, DA or PA) or as predominant patterns (**Supplementary Table S2**).

The NAA MVP was associated with poor prognosis in all patients when applying an optimal cutoff of 0-5% (NAA-) vs >5% (NAA+); (HR 1.42, 95%CI 1.07-1.90, *p*=.016). When stratified by histological subgroup, there was no significant difference in DSS associated with the NAA MVP in patients with LUSC, while for LUAD patients, DSS for the NAA+ subgroup was significantly decreased (HR 1.88, 95%CI 1.24-2.85, *p*=0.003).

**Figure 2:**
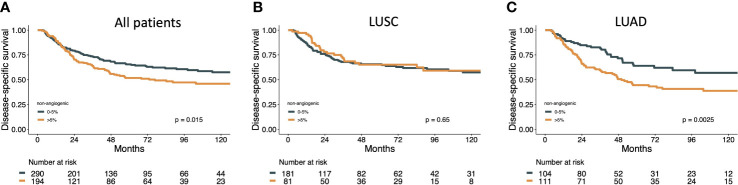
Disease specific survival curves for NAA MVP in **(A)** all patients, **(B)** LUAD and **(C)** LUSC subgroups.

Results of survival analyses for NAA are presented in **Table 1** and **Figure 2**.

Similar associations with poor DSS for the NAA pattern in LUAD patients, not LUSC, was found for other exploratory cutoffs, including none vs any NAA (HR 1.52, 95%CI 0.97-2.36, *p*=0.058) and 0-10% vs >10% NAA (HR 1.70, 95%CI 1.14-2.53, *p*=0.009) as well as for predominant NAA MVP vs angiogenic MVPs (HR 1.65, 1.1-2.48, p=.017) (**Supplementary Table S2**).

**Figure 3:**
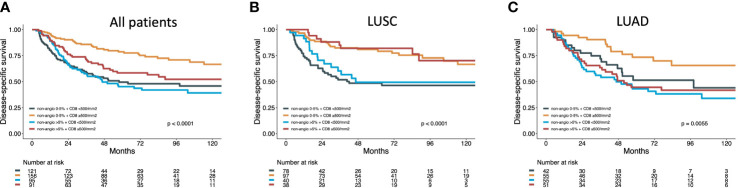
Disease specific survival curves for NAA MVP combined with CD8 density in all **(A)** patients, **(B)** LUAD and **(C)** LUSC subgroups.

Univariate analyses of the prognostic impact of immune cell density combined with NAA MVP status on DSS was assessed in all patients and LUSC and LUAD subgroups. For the LUSC subgroup, high density of CD8_TS contributed to a significantly improved DSS for patients with both NAA+ and NAA- tumors. For LUAD tumors, a high immune cell density added no prognostic impact for NAA+ tumors (**Figure 3**). Similar results are also shown for CD3, CD4, CD45RO, PD1, CD204 (**Supplementary Figure S3**).

### Multivariable analysis

Results from the multivariable Cox regression analysis are presented in **Table 3**. NAA+ MVP was identified as an independent negative prognostic factor for DSS (HR 1.67, 95%CI 1.24-2.24, *p*=.0006). In histological subgroup analysis, the prognostic impact was limited to LUAD patients (HR 2.37 95%CI 1.50-3.73, *p*=.0002). The prognostic impact was significant for LUAD patients also when applying other exploratory cutoffs for NAA (none vs any NAA, HR 1.84, 95%CI 1.14-2.96, *p=*0.012; 0-10% vs >10% NAA, HR 1.78, 95%CI 1.21-2.83, *p*=0.015; predominant NAA vs angiogenic MVPs, HR 2.09, 95%CI 1.34-3.27, *p*=0.001) (**Supplementary Table S3**).

**Table 3:** Results of Cox Regression analysis summarizing significant independent prognostic factors for DSS in all patients and LUSC and LUAD histological subgroups.

	Original model		Model including NAA					
			All patients		LUSC		LUAD	
	HR (95% CI)	*p*	HR (95% CI)	*p*	HR (95% CI)	*p*	HR (95% CI)	*p*
**Sex**		**.0009**		**.028**		.20*		**.035**
female	1		1		1		1	
male	1.67 (1.23-2.26)		1.43 (1.04-1.98)		1.39 (.83-1.35		1.59 (1.03-2.45)	
**Smoking**		**.012**		**.007**		.34		**.016**
never	1		1		1		1	
former	.43 (.22-.85)	.014	.45 (.22-.89)	.022	.53 (.16-1.78)		.37 (.15-.91)	.030
present	.38 (.20-.72)	.003	.35 (.18-.70)	.003	.43 (.13-1.47)		.28 (.12-.68)	.005
**ECOG perf. status**		**.013**		**.004**		**.020**		.087
0	1		1		1		1	
1	1.47 (1.11-1.94)	.007	1.69 (1.24-2.30)	.0009	1.89 (1.2-3.01)	.006	1.56 (1.00-2.43)	.052
2	1.67 (.93-2.99)	.086	1.45 (.75-2.80)	.267	1.77 (.74-4.25)	.198	2.02 (.71-5.78)	.19
**Pathological stage**		**<.0001**		**<.0001**		**<.0001**		**<.0001**
Stage I	1		1		1		1	
Stage II	1.55 (1.10-2.17)	.012	1.5 (1.03-2.18)	.034	1.18 (.69-2.03)	.550	2.00 (1.28-3.39)	.010
Stage IIIA	3.68 (2.61-5.17)	<.0001	3.86 (2.68-5.56)	<.0001	3.97 (2.35-6.70)	<.0001	4.18 (2.50-6.99)	<.0001
**Histological subgroups**		.057		.24*				
LUSC	1		1					
LUAD	1.38 (1.04-1.82)	.025	1.28 (.94-1.74)					
Other	.64 (.15-2.63)	.54	.74 (.18-3.07)					
**Differentiation**		**.012**		**<.001**		**.003**		**.007**
Well	1		1		1		1	
Moderate	1.54 (.93-2.54)		1.55 (.90-2.68)	.12	1.41 (.55-3.61)	.48	1.82 (.88-3.75)	.11
Poor	2.01(1.23-3.30)		2.51 (1.46-4.30)	.0008	2.84 (1.12-7-23)	.029	2.86 (1.43-5.73)	.003
**Vascular infiltration**		**.002**		**.022**		.18*		**.049**
No	1		1				1	
Yes	1.78 (1.28-2.47)		1.62 (1.12-2.35)				1.77 (1.05-3.73)	
**NAA microvessel pattern**		*NE*		**.0006**		.75*		**.0002**
NAA- (0-5%)			1		1		1	
NAA+ (>5%)			1.67 (1.24-2.24)		.93 (.57-1.50)		2.37 (1.50-3.73)	

bold numbers are significant. CI, confidence interval; HR, hazard ratio; DSS, disease-specific survival; ECOG perf. status, Eastern Cooperative Oncology Group performance status; LUAD, lung adenocarcionma; LUSC, lung squamous cell carcinoma; NE, not entered; NSCLC, non-small cell lung carcinoma; *Removed from the model.

## Discussion

In our cohort of resected NSCLC patients, a detailed evaluation of the distribution of angiogenic and non-angiogenic microvessel patterns (MVPs) was performed. Most tumors presented multiple MVPs, and a predominant non-angiogenic alveolar (NAA) MVP was rare (18%). A NAA pattern was more frequently observed in LUAD than LUSC tumors. The NAA pattern was associated with poor prognosis, even when it occupied a limited proportion of the tumor area (>5%, NAA+), in 40% of tumors. In multivariate analysis, the NAA+ pattern was a significant independent negative prognostic factor, and when stratified by histological subgroups, the prognostic effect was limited to patients with LUAD. The immune cell density (CD3, CD4, CD8, CD45RO, PD1, CD204) added prognostic value for all LUSC tumors and in NAA- LUAD tumors. No prognostic effect, however, was found for immune cell density in NAA+ LUAD tumors. In addition, markers associated with metabolism (MCT1, MCT4 and GLUT1) was divergently associated between the four MVPs.

The main weakness of this study is the explorative design. Associations between MVPs and molecular markers were analyzed by multiple testing and without a predefined hypothesis. Adjustment for multiple testing was performed (Bonferroni) in order to avoid type I errors in correlation analyses. Yet, we may describe significant associations but must be careful suggesting causality. Further experimental studies are needed to validate potential causality. Applying an “optimal cut-off” approach for dichotomization of variables in survival analyses introduces a risk of false positive results, but for NAA several cutoffs yielded significant results, in both uni- and multivariate analyses for LUAD. Another potential weakness arises when comparing whole slide scoring (MVPs, TILs) with TMA (other markers) scoring approaches. In our experience, the potential pitfall of tumor heterogeneity is largely avoided when applying mean scores of multiple TMA cores from each tumor (24).

The use of an unselected cohort strengthens the study. Further, an experienced pathologist (FP) in the field of MVPs was in charge of the scoring, and the frequencies of the different MVPs are similar to previous reports (11). Finally, being able to explore associations with a variety of molecular markers previously analyzed in the same tumors is a great asset to the study.

Results from previously published studies reporting the prognostic impact of the NAA pattern in NSCLC have been conflicting. Some studies, however, have indicated that the NAA pattern is associated with a poor prognosis (4, 51, 52). To our knowledge, this is the first study identifying NAA as an independent negative prognostic factor, limited to the LUAD subgroup. As a consequence, one may consider whether patients with LUAD tumors with a NAA MVP are in special need of close follow-up or adjuvant therapy.

This is also the first study to evaluate the combined prognostic impact of MVP and immunological markers. Intriguingly, when a NAA MVP was present (>5%), several immunological markers added prognostic impact in LUSC, but not in LUAD tumors (CD3, CD4, CD8, CD45RO, PD1, CD204). A biological rationale which explains these differences in LUAD vs LUSC can, based on our results, only be speculative. However, a search for immunological biomarkers of prognostic impact for NSCLC patients is ongoing (53, 54). Our group has previously found that immune cells, including CD8, are strong predictors of survival, especially for patients with resected LUSC tumors (16, 18). The present results indicate that some immunological markers, including CD8, may not be of prognostic significance in resected LUAD with NAA+. On the contrary, in NAA- LUAD tumors, a high CD8 has a strong prognostic impact. This may have clinical implications when optimizing the use of CD8 in a resected NSCLC immune score setting.

It has previously been speculated that NAA tumors are less immunogenic compared to DA tumors (4, 5). In line with this hypothesis, we observe several innate and adaptive immune markers with weak associations with a DA MVP. In contrast, CD8, CSF1R and CD138 are weakly inversely correlated to a NAA+ MVP (12). However, after adjustment for multiple testing these results are not significant. Hence, further investigations may improve our understanding of the immunogenic status in NAA MVP. In light of the above-mentioned lack of prognostic impact of immune cells in LUAD NAA+, one may speculate that neither angiogenesis inhibition nor immunotherapy are optimal treatment strategies in NAA+ LUAD patients.

Interestingly, some of the stronger significant associations were between markers related to metabolism (MCT1, MCT4 and GLUT1) and MVPs. Notably, significant positive correlations are found only with the BA pattern. In hypoxic conditions, a metabolic adaption to produce energy by glycolysis occurs in normal cells (42). Cancer cells, on the other hand, seem to prefer glycolysis despite the presence of oxygen (Warburg effect) (42). In this complex process, lactate and proton transmembrane symporters, MCT1 and MCT4, are pivotal. They prevent intracellular acidification and shuffle the lactate as an energy substrate for the respiratory chain in more oxygenated tissue (42). Although we simply observe associations, it is tempting to hypothesize that glycolysis and degree of hypoxia in the tumor microenvironment, or at least the regulation of these metabolic markers, differs significantly between MVPs. The positive correlation between GLUT1 and MCT1 and negative MCT4 correlation with BA pattern points to a very metabolic active tissue as GLUT1 and MCT1 are energy importers. However, as there is a tendency (though not significant when adjusted for multiple testing) towards an association with CAIX (endogen hypoxic marker) the BA tissue may be more hypoxic. This seems intuitive in a morphological sense, as the distance between vessels is often greater than in the other MVPs (as illustrated by an inverse correlation with microvessel density, MVD). From a clinical point of view, hypoxic tumors are known to be less sensitive to radiotherapy. Hence, it would be interesting to explore whether there is a relationship between metabolic markers, MVPs and radiotherapy response.

In conclusion, NSCLC tumors display different MVPs and several patterns often coexist. NAA+ MVP is an independent negative prognostic factor in LUAD, but not LUSC tumors. Several immunological markers add prognostic impact in LUSC and LUAD tumors with NAA- MVP, but not in LUAD with NAA+ MVP. Further investigation is needed to interpret these novel results as we need further knowledge regarding the relationship between tumor characteristics and AI and IO efficiency.

Our next step is to validate the prognostic impact of MVPs in combination with immunological markers in our comprehensive multicenter prospective NSCLC trial (NCT03299478). A predefined, potentially digital, scoring approach is being considered, as well as including gene expression and mutation data in multivariate analyses. Further studies are needed to increase biological understanding and to potentially establish MVPs, in combination with immunological status, as clinically implementable predictive factors in NSCLC treatment.

## Data availability statement

Most relevant data supporting the conclusions of this article are included in Supplementary Figures/Tables. The complete datasets will be made available by the authors upon reasonable request. Requests to access the datasets should be directed to Tom Donnem (tom.donnem@uit.no).

## Ethics statement

The studies involving human participants were reviewed and approved by Northern Norway Regional Ethical Committee. Written informed consent for participation was not required for this study in accordance with the national legislation and the institutional requirements.

## Author contributions

E-EP, FP, and TD conceptualized and designed the study. E-EP, SA, MR, MIP, AL, MP, TK, L-TB, FP and TD were responsible or contributed to acquisition, analysis, or interpretation of data. E-EP and MR performed the statistical analysis. MIP and AL provided technical support. TD obtained funding. E-EP and TD drafted the manuscript. All authors contributed to the article and approved the submitted version.
