# Supplementary material for: Impact of microvessel patterns and immune status in NSCLC: a non-angiogenic vasculature is an independent negative prognostic factor in lung adenocarcinoma
Source: Front Oncol. 2023 Apr 26;13:1157461. doi: 10.3389/fonc.2023.1157461 (PMC10169734; doi:10.3389/fonc.2023.1157461)
Supplement: Supplementary file 1 [file DataSheet_1.pdf]

Supplementary Figure 1: Distribution of microvessel patterns.

A) Frequency by percent MVPs in each tumor (several MVPs may coexist in each tumor).

B) Predominant MVP, percent of all scored tumors (one predominant MVP per tumor).

*Null percent score not included in frequency tables

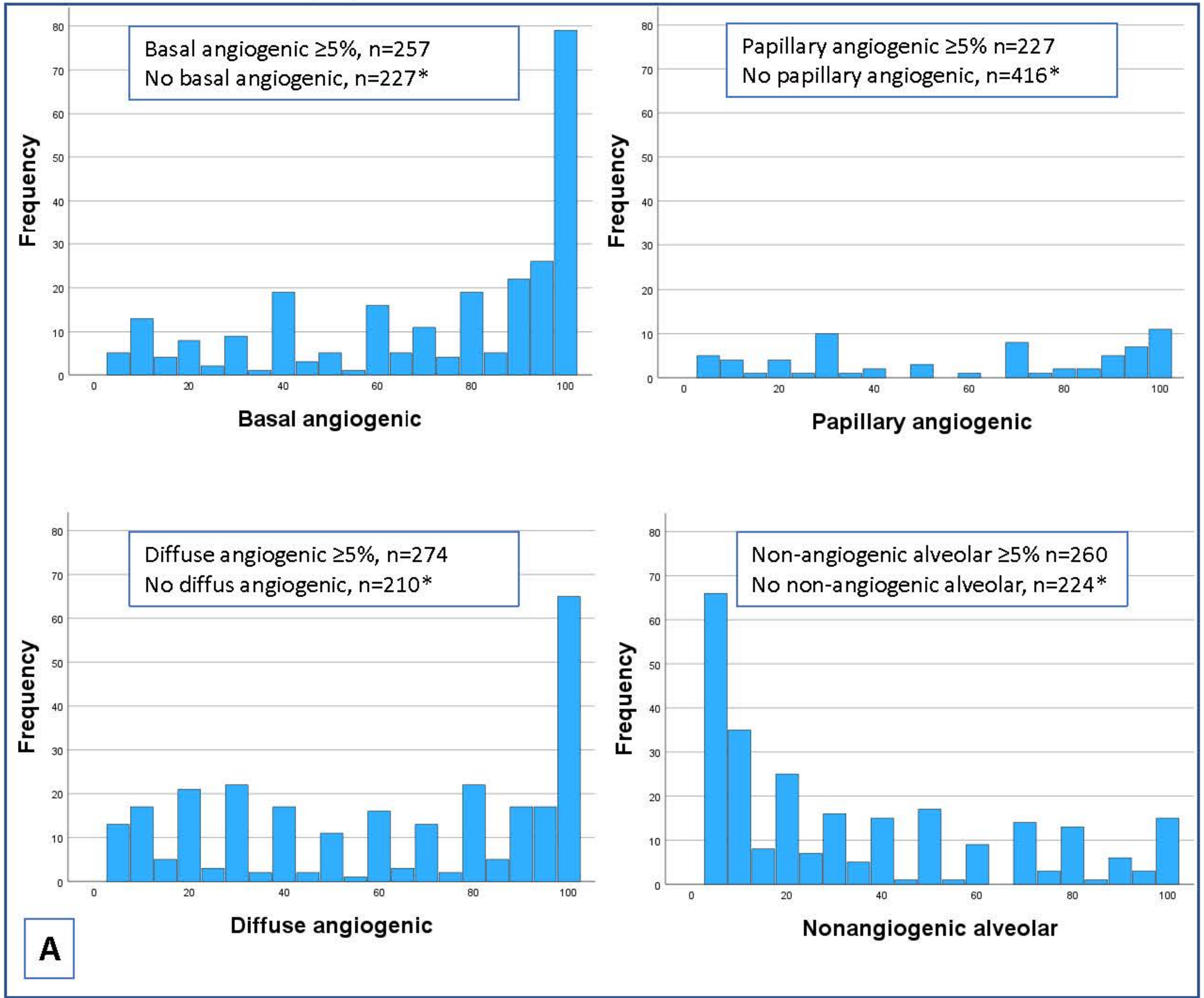

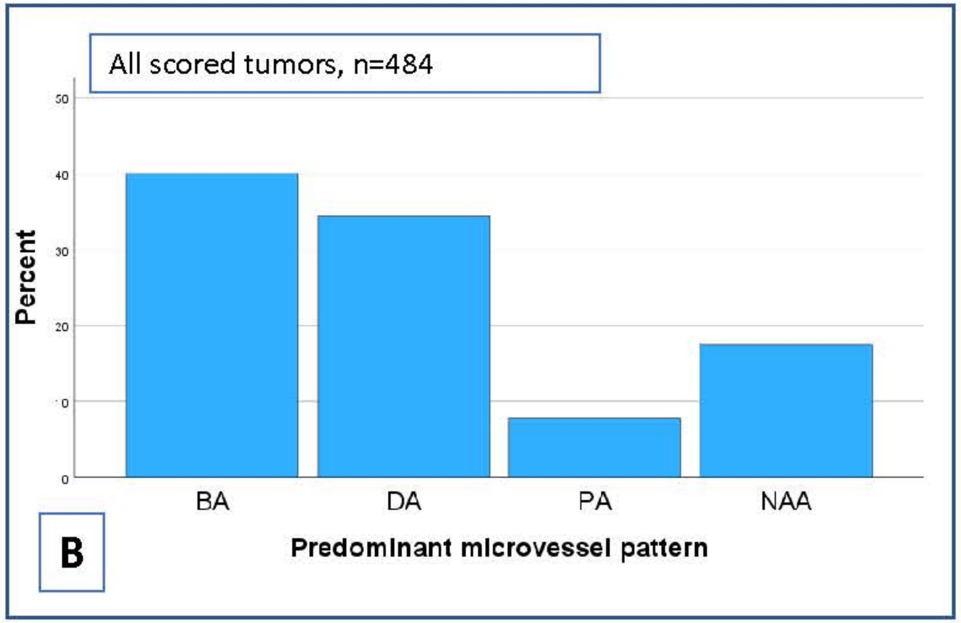

BA, basal angiogenic; DA diffuse angiogenic, PA papillary angiogenic; NAA nonangiogenic alveolar
