# Supplementary material for: Impact of microvessel patterns and immune status in NSCLC: a non-angiogenic vasculature is an independent negative prognostic factor in lung adenocarcinoma
Source: Front Oncol. 2023 Apr 26;13:1157461. doi: 10.3389/fonc.2023.1157461 (PMC10169734; doi:10.3389/fonc.2023.1157461)

Supplementary Figure 2

Correlation matrices illustrating associations between microvessel patterns (MVPs) and markers involved in tumor immunity (n=22), angiogenesis (n=30) or hypoxia/metabolism (n=14). Markers evaluated in tumor epithelial (_T), stromal (_S) compartment, or both combined (_TS). Spearman's ranked correlations.

Immunity_T:

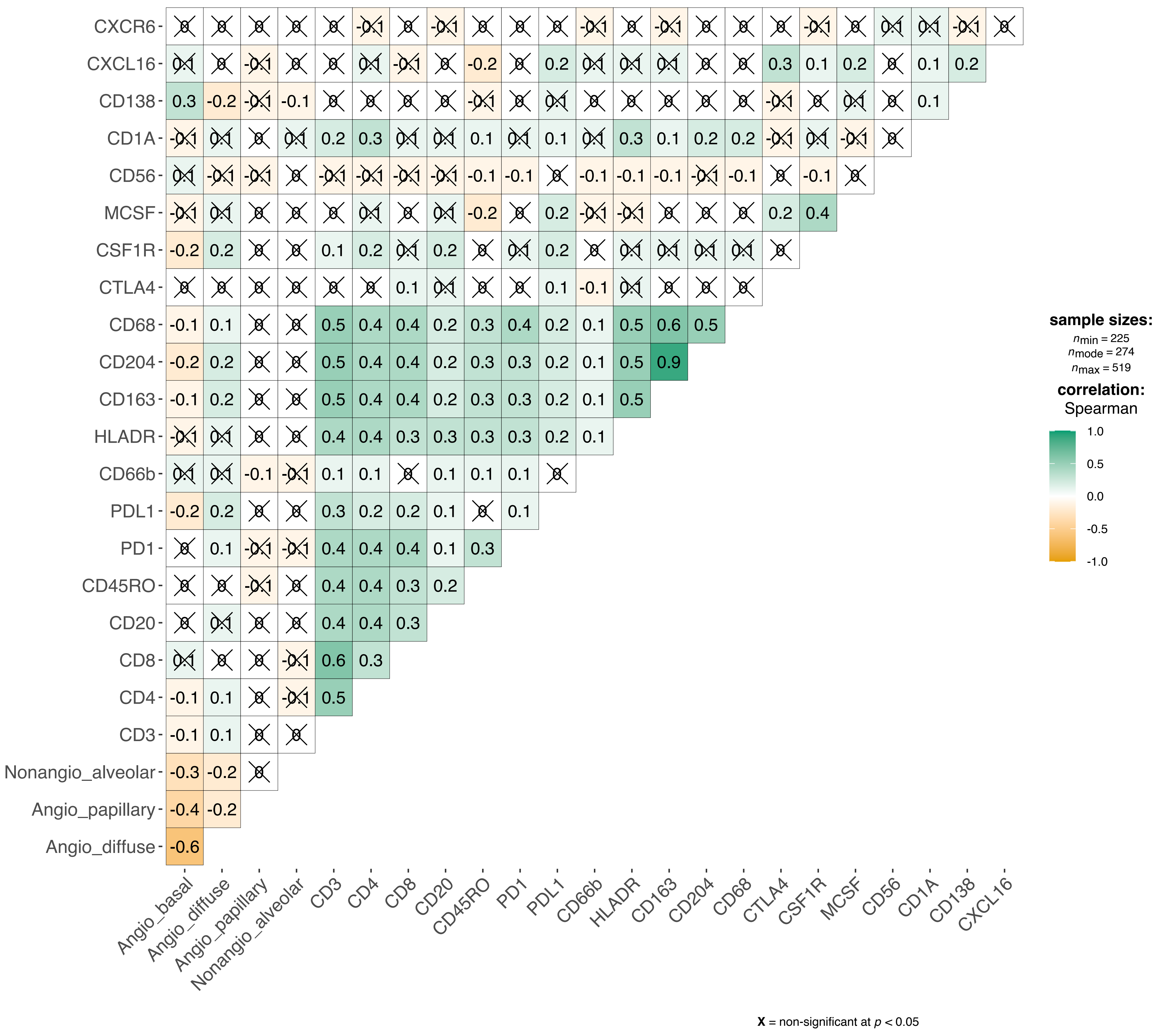

Immunity_S:

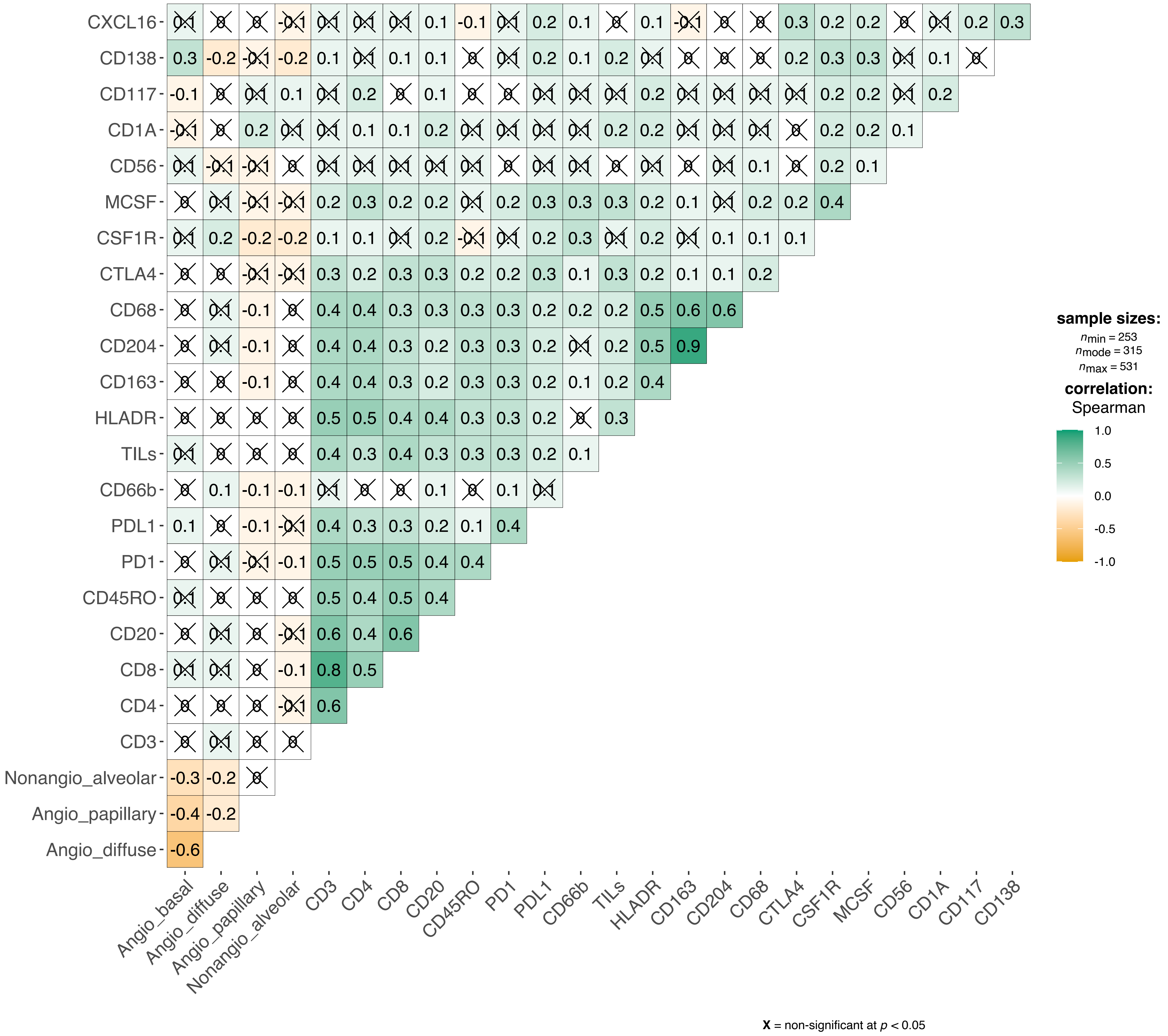
CXCL16
CD138
CD117
CD1A
CD56
MCSF
CSF1R
CTLA4
CD68
CD204
CD163
HLADR
TILs
CD66b
PDL1
PD1
CD45RO
CD20
CD8
CD4
CD3
Nonangio_alveolar
Angio_papillary
Angio_diffuse
Angio_basal
sample sizes:
n_{min} = 253
n_{mode} = 315
n_{max} = 531
correlation:
Spearman
1.0
0.5
0.0
-0.5
-1.0
X = non-significant at $p < 0.05$

Immunity_TS:

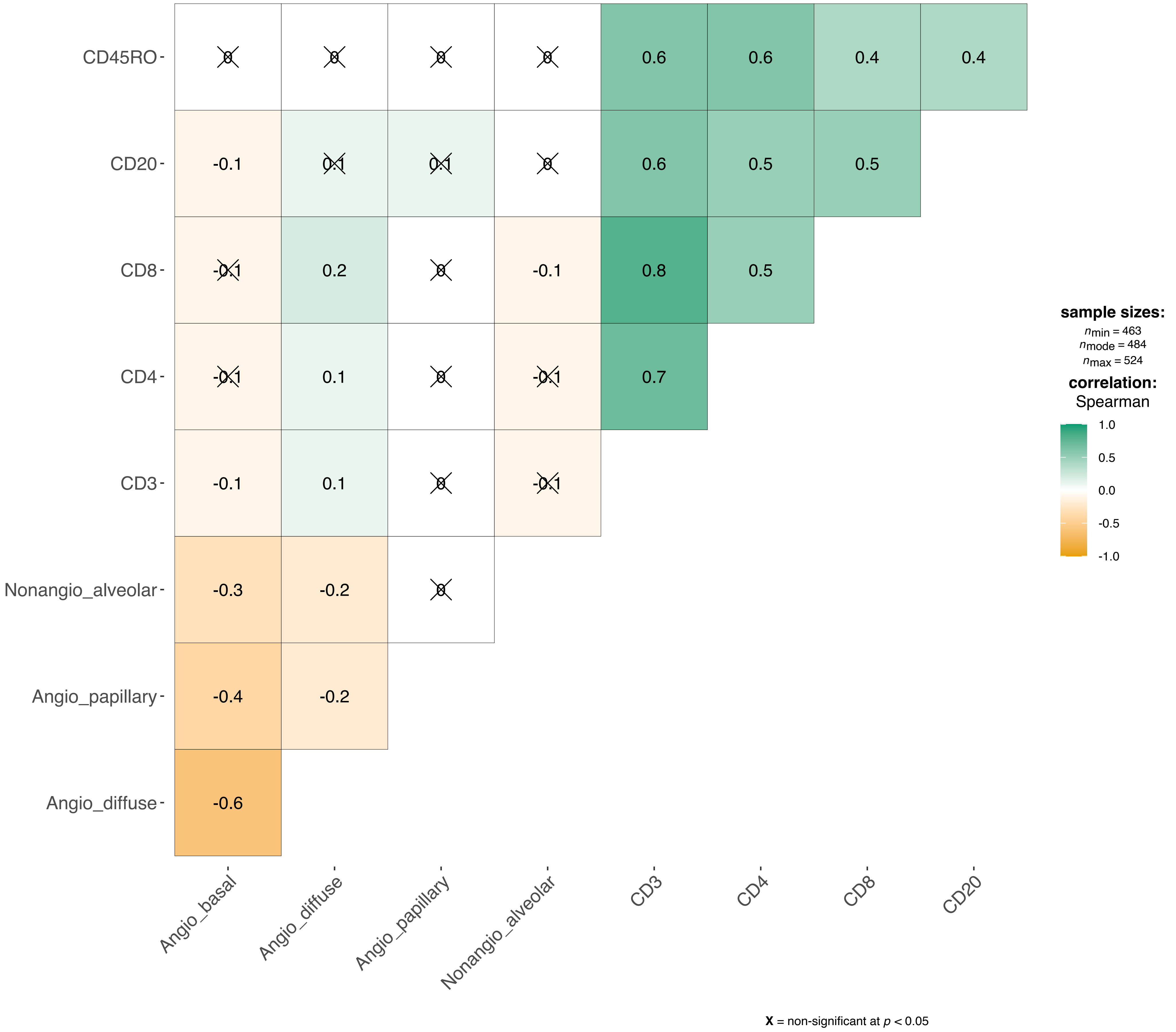

CD45RO
CD20
CD8
CD4
CD3
Nonangio_alveolar
Angio_papillary
Angio_diffuse
Angio_basal
Angio_diffuse
Angio_papillary
Nonangio_alveolar
CD3
CD4
CD8
CD20
sample sizes:
nmin = 463
nmode = 484
nmax = 524
correlation:
Spearman
1.0
0.5
0.0
-0.5
-1.0
X = non-significant at p < 0.05

Angiogenesis_T:

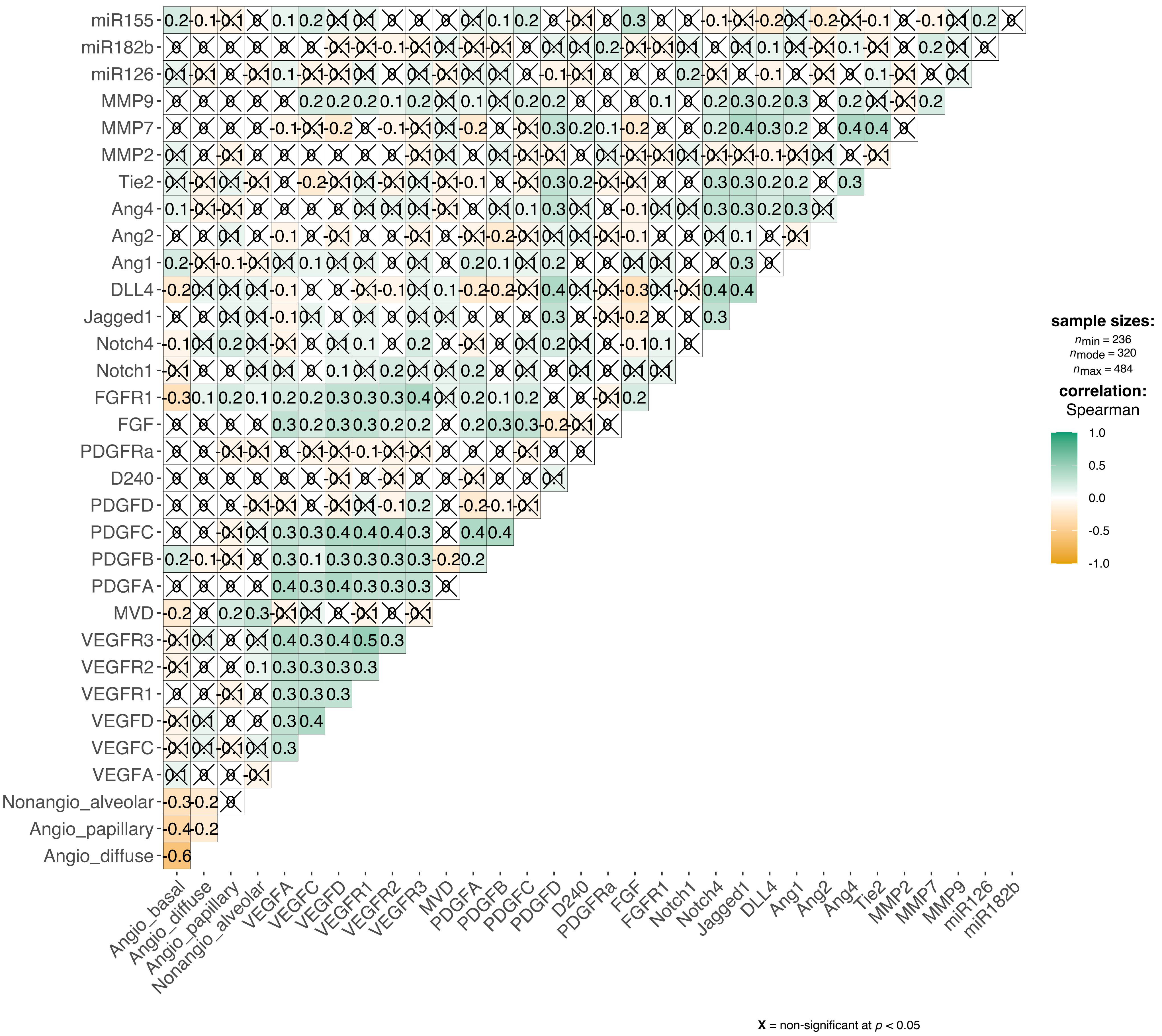

Angiogenesis_S:

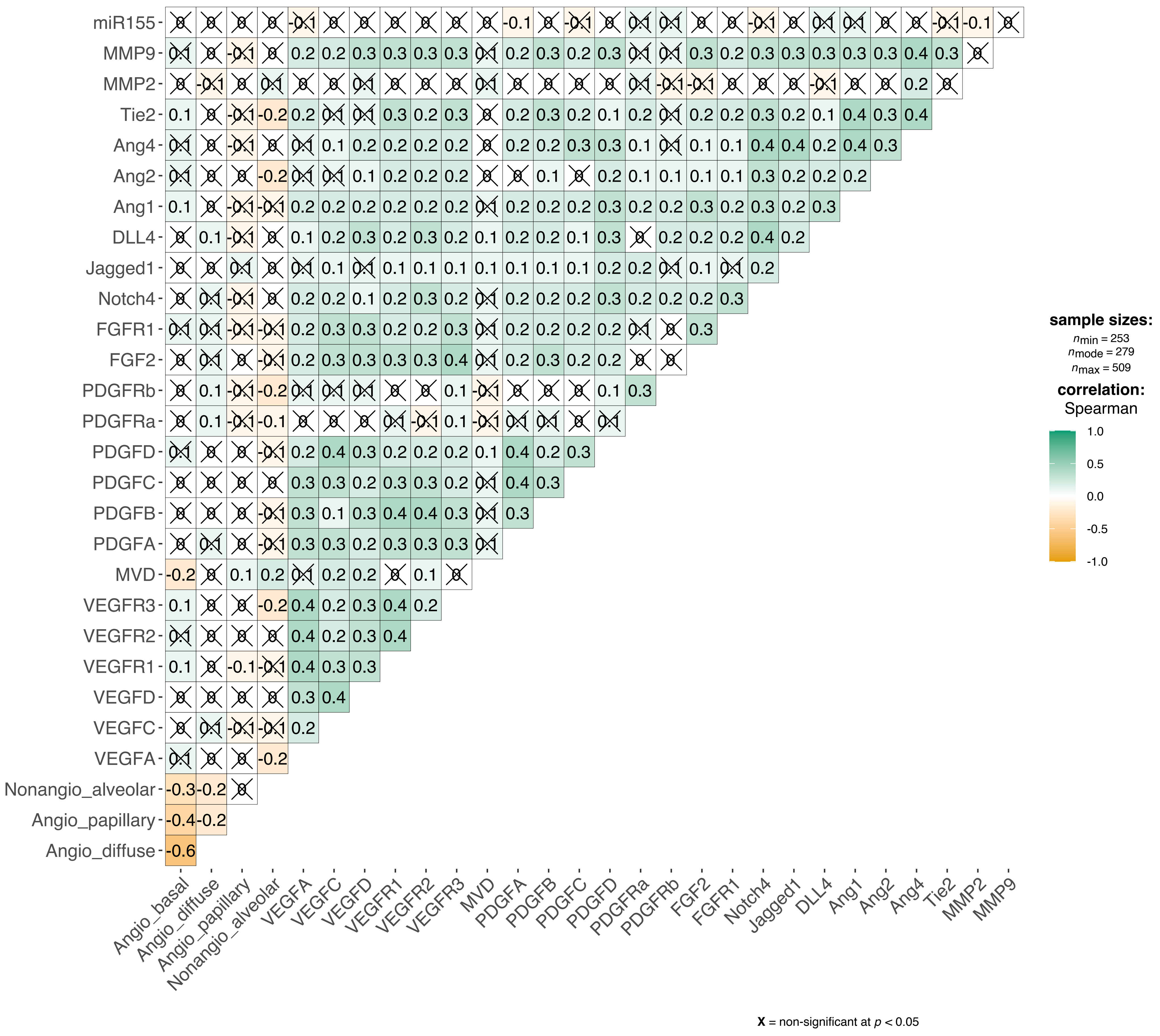

Hypoxia/metabolism_T:

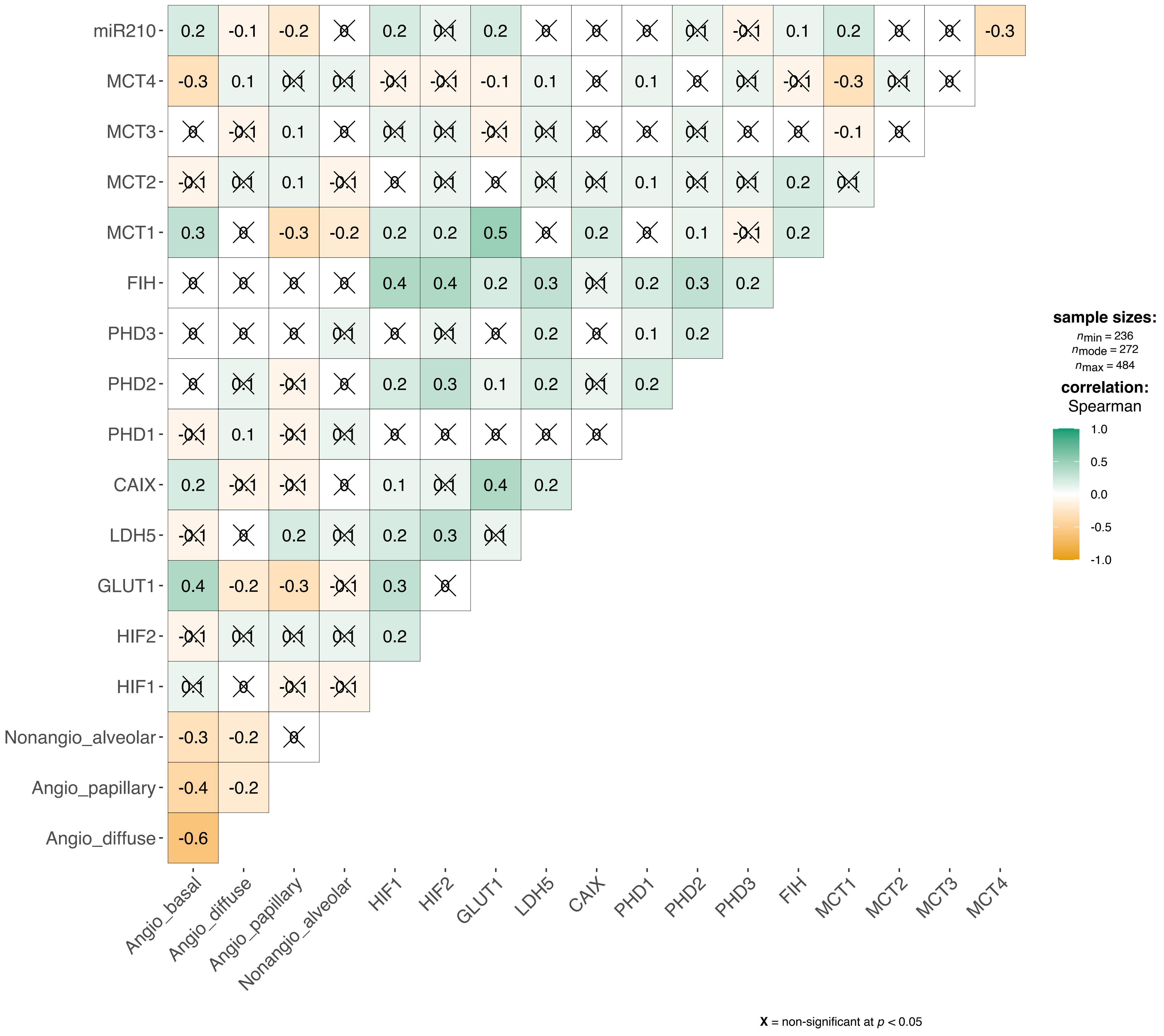

Hypoxia/metabolism_S

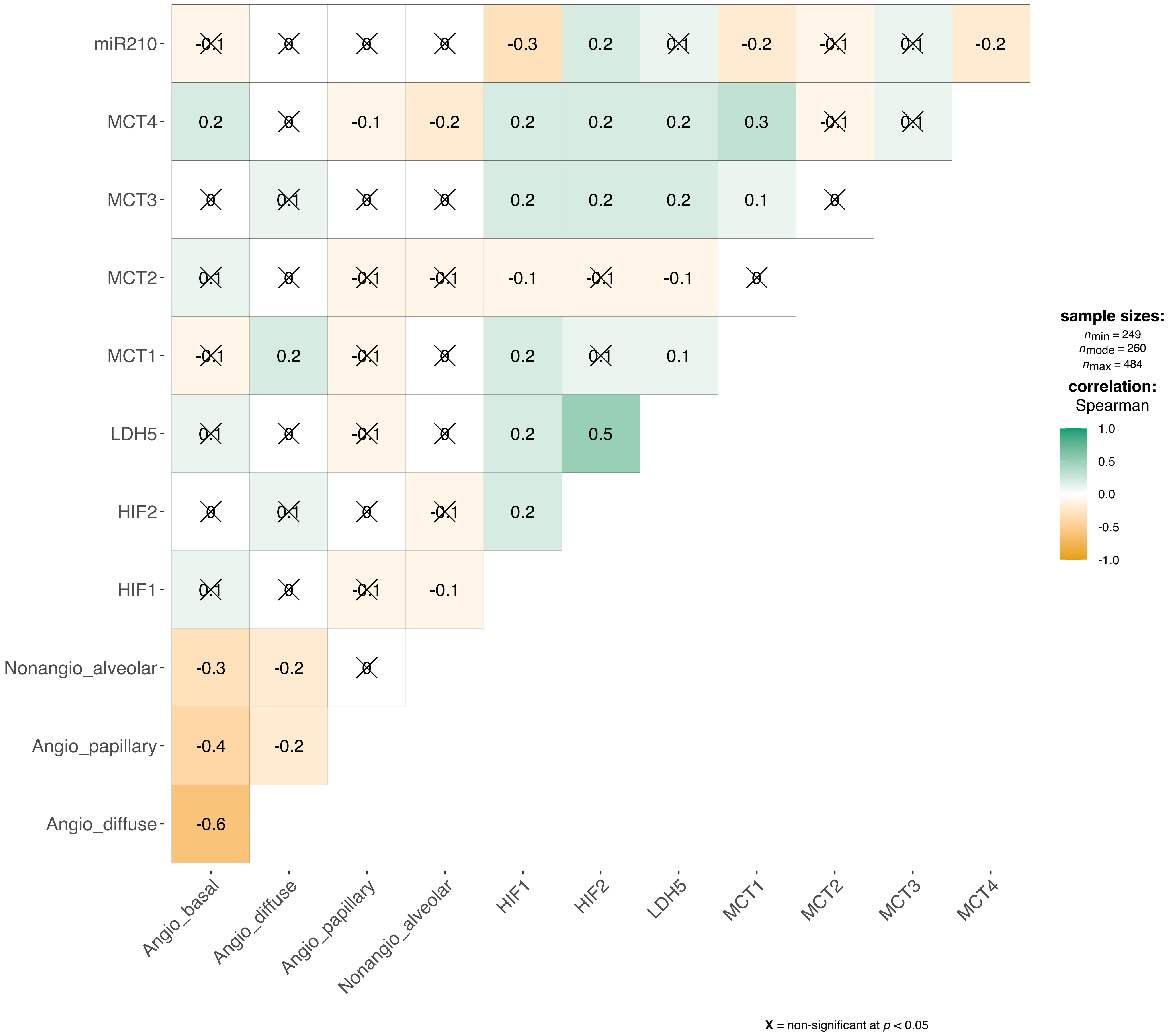

Supplement: Supplementary file 2 [file DataSheet_2.pdf]
