# Supplementary material for: Impact of microvessel patterns and immune status in NSCLC: a non-angiogenic vasculature is an independent negative prognostic factor in lung adenocarcinoma
Source: Front Oncol. 2023 Apr 26;13:1157461. doi: 10.3389/fonc.2023.1157461 (PMC10169734; doi:10.3389/fonc.2023.1157461)
Supplement: Supplementary file 3 [file DataSheet_3.pdf]

Supplementary Figure 3:

Disease specific survival curves for NAA MVP combined with A) CD3_TS, B) CD4_TS, C) CD45RO_TS, D) PD1_T, E) CD204_T for all patients, LUSC and LUAD histological subgroups.

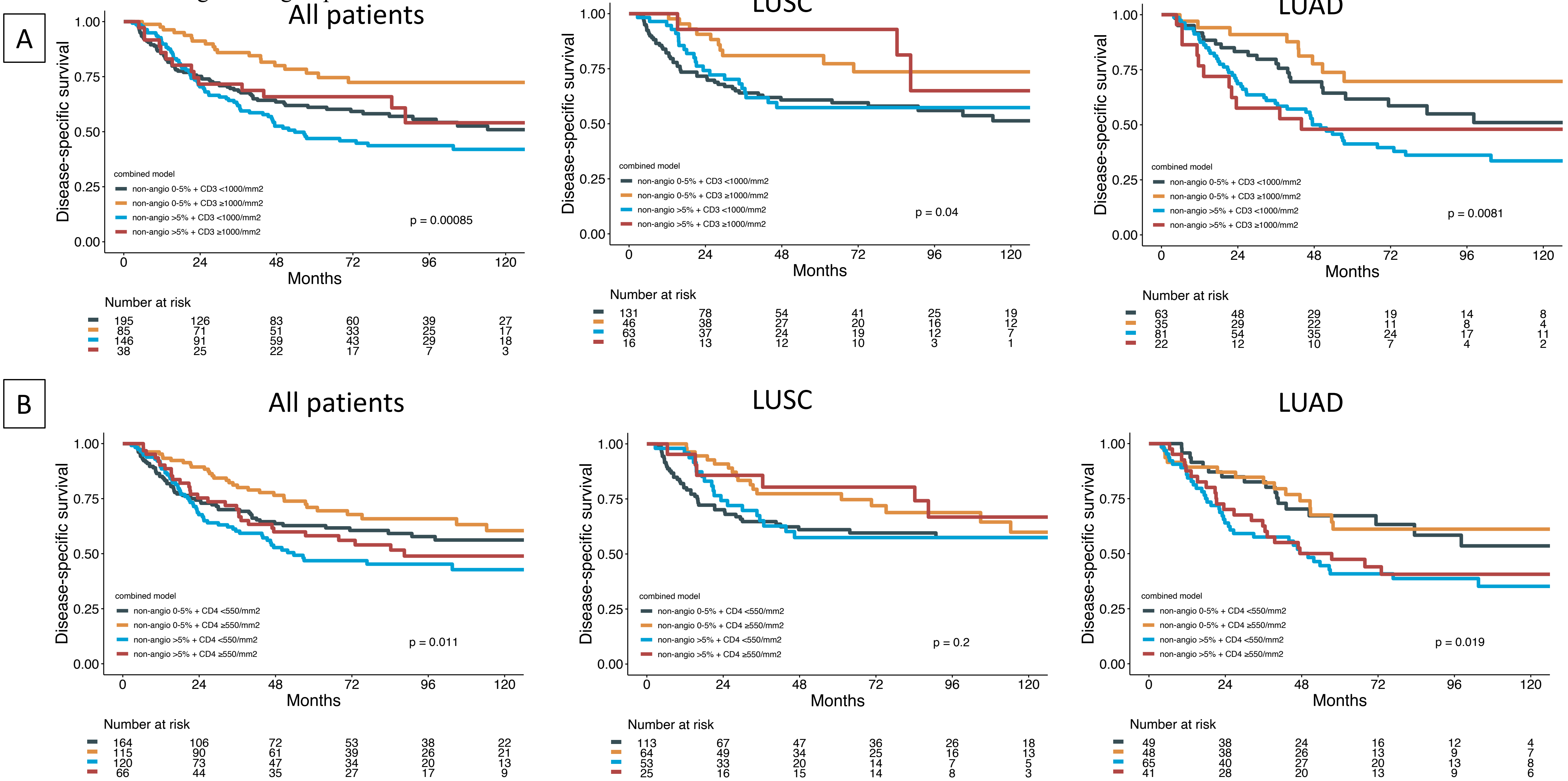

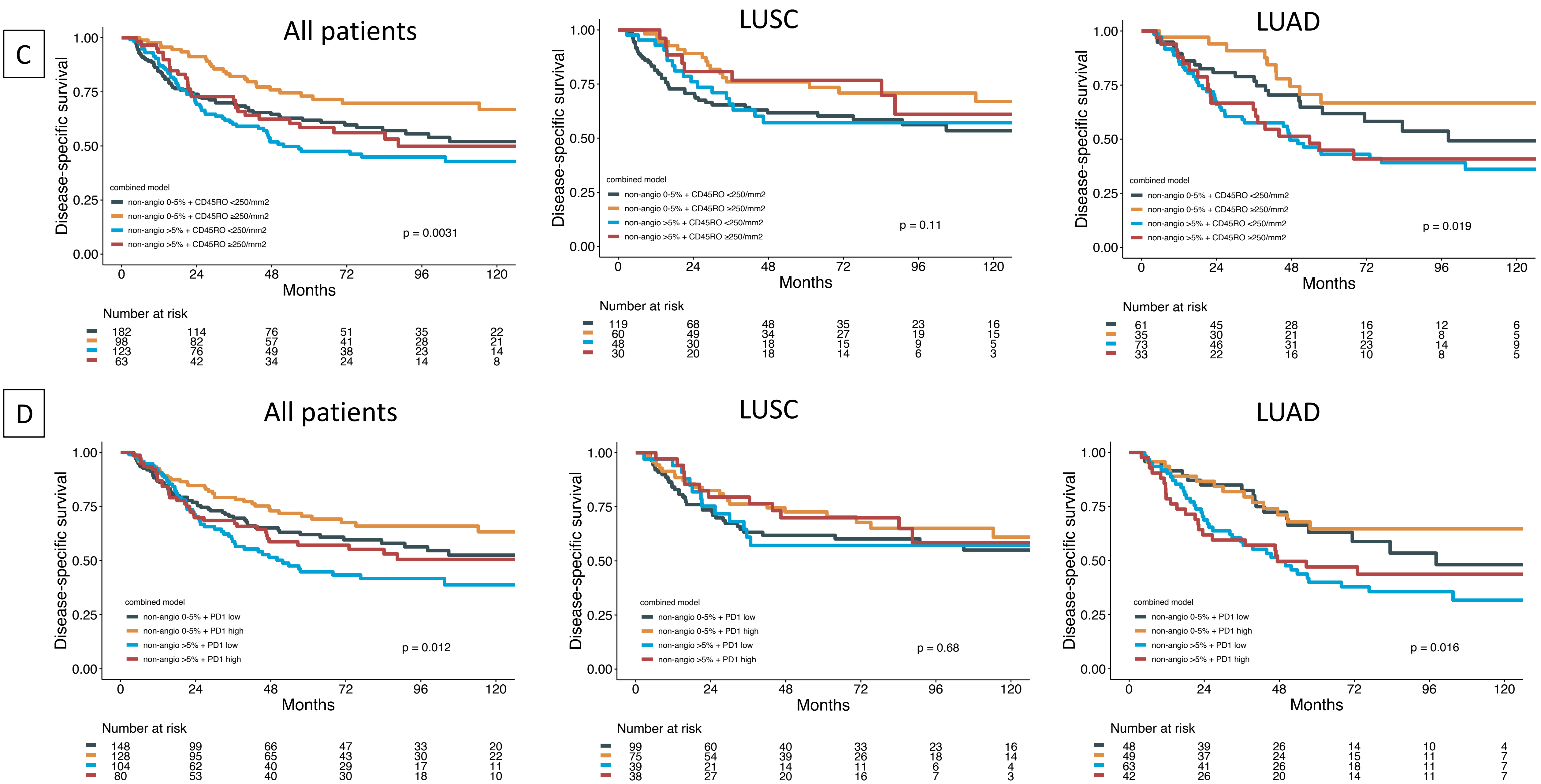
C
All patients
Disease-specific survival
1.00
0.75
0.50
0.25
0.00
combined model
non-angio 0-5% + CD45RO <250/mm2
non-angio 0-5% + CD45RO ≥250/mm2
non-angio >5% + CD45RO <250/mm2
non-angio >5% + CD45RO ≥250/mm2
p = 0.0031
0 24 48 72 96 120
Months
Number at risk
182 114 76 51 35 22
98 82 57 41 28 21
123 76 49 38 23 14
63 42 34 24 14 8
LUSC
p = 0.11
Number at risk
119 68 48 35 23 16
60 49 34 27 19 15
48 30 18 15 9 5
30 20 18 14 6 3
LUAD
p = 0.019
Number at risk
61 45 28 16 12 6
35 30 21 12 8 5
73 46 31 23 14 9
33 22 16 10 8 5
D
All patients
combined model
non-angio 0-5% + PD1 low
non-angio 0-5% + PD1 high
non-angio >5% + PD1 low
non-angio >5% + PD1 high
p = 0.012
Number at risk
148 99 66 47 33 20
128 95 65 43 30 22
104 62 40 29 17 11
80 53 40 30 18 10
LUSC
p = 0.68
Number at risk
99 60 40 33 23 16
75 54 39 26 18 14
39 21 14 11 6 4
38 27 20 16 7 3
LUAD
p = 0.016
Number at risk
48 39 26 14 10 4
49 37 24 15 11 7
63 41 26 18 11 7
42 26 20 14 11 7

E

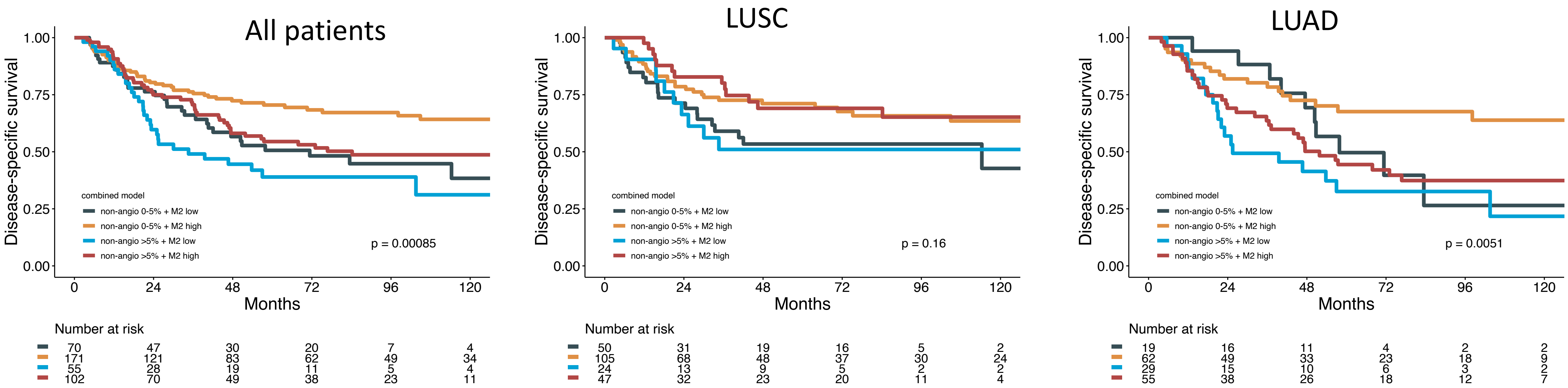
All patients
LUSC
LUAD
Disease-specific survival
1.00
0.75
0.50
0.25
0.00
Months
0
24
48
72
96
120
combined model
non-angio 0-5% + M2 low
non-angio 0-5% + M2 high
non-angio >5% + M2 low
non-angio >5% + M2 high
p = 0.00085
p = 0.16
p = 0.0051
Number at risk
70 47 30 20 7 4
171 121 83 62 49 34
55 28 19 11 5 4
102 70 49 38 23 11
50 31 19 16 5 2
105 68 48 37 30 24
24 13 9 5 2 2
47 32 23 20 11 4
19 16 11 4 2 2
62 49 33 23 18 9
29 15 10 6 3 2
55 38 26 18 12 7
